# Modeling the effect of improved sewage disposal rates on ecological status for aquatic organisms in Japan

**DOI:** 10.1016/j.heliyon.2023.e20943

**Published:** 2023-10-16

**Authors:** Toyohiko Nakakubo, Midori Kawabata, Yuriko Ishikawa, Yuichi Iwasaki

**Affiliations:** a Division of Sustainable Energy and Environmental Engineering, Graduate School of Engineering, Osaka University, 2-1 Yamadaoka, Suita, Osaka 565-0871, Japan; b Cooperative Major in Human Centered Engineering, Graduate School of Humanities and Sciences, Ochanomizu University, 2-1-1 Otsuka, Bunkyo-ku, Tokyo 112-8610, Japan; c National Institute of Advanced Industrial Science and Technology (AIST), 16-1 Onogawa, Tsukuba, Ibaraki 305-8569, Japan

**Keywords:** Onsite wastewater treatment system, Sewage line connection, Scenario analysis, Organic contamination, Macroinvertebrate

## Abstract

Improving sewage disposal rates is an important policy for maintaining the health of aquatic organisms in river environments. In Japan, the rate is not yet 100 %. Two measures are necessary to eliminate the discharge of untreated greywater: (1) increase the number of households connected to sewage lines in areas with sewage systems, and (2) replace single-type household onsite wastewater treatment systems (OWTSs) with combined-type systems. To estimate the effect of improving the disposal rate on river water quality, we developed a hydrology-based organic pollution assessment model with a gridded spatial resolution of 250 m to estimate the biochemical oxygen demand (BOD) in rivers in Gunma Prefecture, Japan. We considered three scenarios based on the sewage disposal rate of 70.5 % in 2015. In Scenario A, the disposal rate is increased to 75.2 % in 2030 by increasing the connection rate to sewage lines. In Scenario B, the rate is increased to 88.2 % in 2030 through additional progress in converting from single-to combined-type OWTSs. In Scenario C, the rate reaches 100 % by 2040. The ecological status of rivers was evaluated using taxon richness of *Ephemeroptera*, *Plecoptera*, and *Trichoptera* estimated from its reported relationship to BOD. The number of sites in Gunma Prefecture polluted by organic waste classified as III (poor) and IV (very poor) was estimated to be 1610 under the present state (2015) and decreased to 1212 (25 % reduction) in Scenario A, 619 (62 % reduction) in Scenario B, and 50 (97 % reduction) in Scenario C, with the improvements mainly in small branch rivers. The effects of improved disposal rates were mainly evident in areas with relative high population densities using single-type OWTSs outside of areas with a sewage system, and measures taken in these areas were shown to be effective.

## Abbreviations

EPT: *Ephemeroptera*, *plecoptera*, and *trichoptera*
GWF: Greywater footprint
OWTS: Onsite wastewater treatment system
RCSF: Rural community sewage facility
WWTP: Wastewater treatment plant

## 1 Introduction

The establishment of a well-designed wastewater treatment system to minimize environmental impacts requires the treatment of household greywater. The sustainable development goals, SDGs, set by the United Nations in 2015 [1] include Goal 6.3: “By 2030, improve water quality by reducing pollution, eliminating dumping and minimizing release of hazardous chemicals and materials, halving the proportion of untreated wastewater and substantially increasing recycling and safe reuse globally.” with indicator 6.3.1: “Proportion of domestic and industrial wastewater flows safely treated.” This allows each country to reconfirm the adequacy of its domestic wastewater treatment systems. The main types of treatment systems are a centralized one, with sewage lines connecting to a wastewater treatment plant (WWTP) in densely populated areas, and onsite wastewater treatment systems (OWTSs). In Japan there are still problems with both systems and inadequate treatment of domestic wastewater. For areas with a sewage system, sewage lines are constructed by the public sector, whereas the drainage pipes on private land that connect to the sewage line have to be installed by the resident under cost-sharing [2]. This leads to the issue of unconnected households where only night soil is collected by a municipal enterprise and greywater is discharged directly to a drainage channel.

For OWTSs, the installed components are much like a septic tank, which uses anaerobic biological treatment to promote partial digestion of retained organic matter [3]. Conventional septic systems have two main components: a septic tank and a drain-field (soil absorption layer), in which septic-tank effluent is distributed and treatment functions are achieved through sorption, volatilization, and aerobic degradation [4]. In contrast, Japanese OWTSs have an aerobic septic tank with a leading anaerobic filter zone that separates solid matter, stores sludge, and degrades organic matter by anaerobic microorganisms, and a subsequent contact aeration zone, where aerobic microorganisms degrade organic matter and oxidize ammonia. With the application of aerobic biological treatment, outlet water is discharged directly to a drainage channel and soil filtration is not used. Two types of OWTSs are installed in Japan. The combined type accepts both toilet water and greywater, and the single type receives only toilet water and greywater is discharged directly without treatment. Although new installations of single-type systems were banned in fiscal year (FY) 2001, replacing them with combined-type tanks was a voluntary decision left to the citizens. As a result, of the total 7,517,947 tanks installed in Japan, 3,639,887 (48.4 %) of the single type remained in FY2020 [5]. One of the main tasks to accomplish in the field for protecting aquatic environments is eliminating the direct discharge of greywater from both areas with sewage systems and areas with OWTSs.

Japanese development project of domestic wastewater treatment systems is managed based on sewage treatment population penetration rate as a policy indicator, and the rate has reached 92.1 % as of FY2020 on a national average [6]. The numerator of this indicator includes the population living in areas with sewage systems and those using a combined-type OWTS, and then population unconnected to sewage lines is also counted. OECD [7] shows the secular change of sewage treatment connection rates (the percentage of the population actually connected to a WWTP, not taking into account independent private facilities) in mainly developed countries, and the data in Japan is 80.1 % in 2020. The problem is that the Japanese policy indicator has not adequately considered the unconnected population for centralized systems. On the other hand, when the penetration rates in FY2020 are aggregated by municipality population size, the average for municipalities with populations of 50,000 to 100,000 is 87.1 %, and the same value for less than 50,000 is 81.9 % [6]. For small and medium-sized municipalities, the population using a single-type OWTS, not counted as treatment one, leads to a lower level of penetration.

One approach to evaluating the environmental burden from water pollution in river basins is the greywater footprint (GWF), defined as the volume of freshwater required to assimilate the pollutant load and based on natural background concentrations and existing ambient water-quality standards [8]. Mekonnen & Hoekstra [9] conduced a global GWF assessment at a spatial resolution of 5 × 5 arc minutes; the GWF was calculated by dividing the nitrogen (N) load by the difference between the ambient water-quality standard for N and the natural concentration of N in the receiving water body. The N load from households to surface water was estimated by considering the fraction of the total population connected to a public sewage system, and the environmental pressure from untreated household wastewater was also covered. Feng et al. [10] quantified the GWF in China from 2003 to 2018 on the basis of four pollutants: chemical oxygen demand (COD), ammonia nitrogen (NH_3_–N), total nitrogen (TN), and total phosphorus (TP). In their study, the wastewater collection and treatment rates reflected the pollution load from urban residents, and they similarly considered the proportion of the rural households using dry or flushing toilets. They showed that among the pollution sources, urban and rural residents made a relatively large contribution to GWF via NH_3_–N. For household wastewater, the presence of untreated toilet pollutants dominated the assessment of GWF quantified by TN and NH_3_–N. Instead of N, organic pollution was adopted for the assessment and biochemical oxygen demand (BOD) was used as the indicator to trace untreated greywater; N is mainly found in toilet discharge.

Greywater has a potential to be reused onsite through simple treatment in terms of its physical, chemical, and microbial characteristics. Vuppaladadiyam et al. [11] summarized treatment technologies, reuse standards and water quality requirements for greywater reuse in view of trends in various countries. Oteng-Peprah et al. [12] organized the physicochemical and biological characteristics of greywater by classifying them into low-income countries and high-income ones, and greywater reuse strategies were discussed, with a particular focus on efforts in developing countries. Shaikh & Ahammed [13] focused on classifying the reuse of greywater into light greywater and dark greywater, and analyzed the variation in water quality for the classified one. This study mainly targets appropriate treatment of greywater and improvement effect on stream-water organic pollution. There are cases where reuse is being considered from the status where untreated greywater is discharged, and there is a need to develop a method to analyze the effect of water quality improvement in river basins.

Benthic macroinvertebrates in rivers, such as mayflies and stoneflies, have been utilized as indicators of environmental degradation [[14], [15], [16]]. Although many macroinvertebrate metrics have been proposed and employed in environmental assessments, the number of taxa belonging to the orders *Ephemeroptera*, *Plecoptera*, and *Trichoptera* (EPT richness) is a commonly used metric [14,16]. The variation in EPT richness has been reported to follow changes in ecosystem processes, such as leaf-litter processing and invertebrate secondary production [17]. Several studies have examined the relationships between organic contamination levels, as indicated by BOD, and EPT richness (e.g., Refs. [18,19]), and such a relationship has been quantified in Japanese rivers [20]. However, no attempt has been made to predict changes in EPT richness as an indicator of river ecosystem health by modeling improvements in sewage disposal rates.

We focused on the possibility of determining changes in stream ecological status via a macroinvertebrate-related index by reducing direct discharge of greywater from households in watershed areas of Japan. The objectives of this research were to (1) design scenarios for improving sewage disposal rates by connecting households to sewage lines in areas with sewer systems, and by replacing single-type OWTSs with the combined type outside of these areas; (2) estimate stream BOD through application of an organic pollution model; and (3) evaluate the effects of these improvements on stream water-quality classification based on the potential for inhabitation by EPT.

## 2 Material and methods

### 2.1 Basic conditions in the targeted river basin

**Fig. 1:**
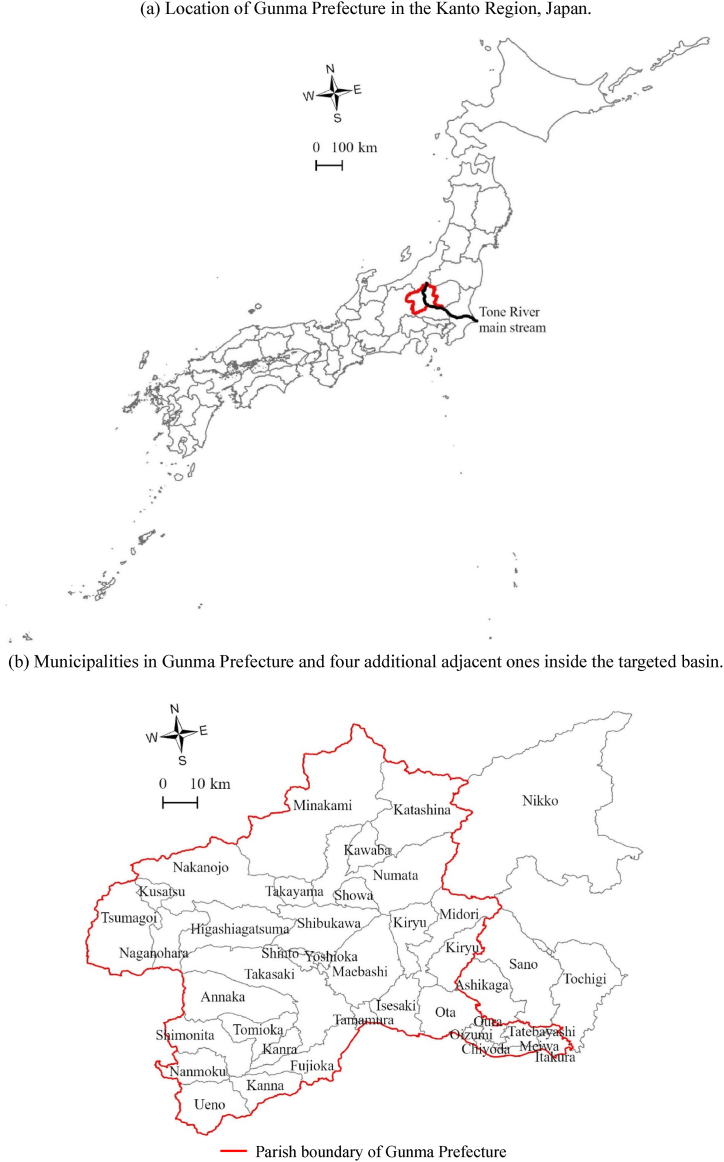

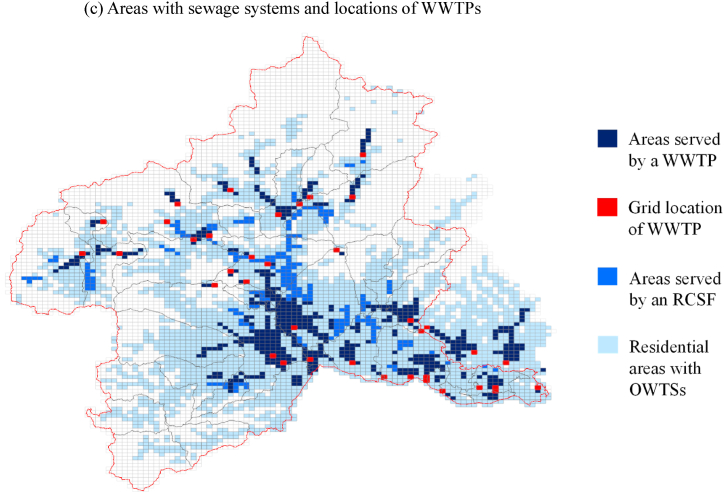
Basic wastewater treatment conditions in the targeted area in Gunma Prefecture, showing (a) location, (b) municipalities and (c) urban wastewater infrastructure. Spatial resolution in (c) is 1 × 1 km.

The Tone River basin has a catchment area of 16,840 km^2^ across six prefectures in the Kanto region of Japan. For this study, we targeted the upstream area in Gunma Prefecture, mapped in Fig. 1 (1a: location in Japan, 1b: individual municipalities, and 1c: areas with sewage systems). Small and medium-sized rivers in Gunma Prefecture were included for evaluation, and an additional four neighboring municipalities were included within the study area for BOD input, based on their contributions to the target rivers. This region was selected for two reasons: (1) sewage disposal rates were determined at a prefecture scale, and the rate in Gunma Prefecture was the lowest in the Kanto region in FY2015 [21]; and (2) the BOD burdens from household, commercial, and industrial wastewater from outside the prefecture were negligible because of its location in the upper part of the drainage basin, so there is no need to set background river concentrations when applying a model simulation.

We prepared a mesh model for the areas with sewage lines at a gridded spatial resolution of 1 km using the related map provided by the sewerage division of the Gunma Prefectural Government. We used ArcGIS Pro (Esri Inc., Redlands, California, USA) as a space analysis tool. Aside from areas with a sewage system connected to a WWTP, some agricultural villages have sewage lines that connect households to a rural community sewage facility (RCSF). A map of the Japanese population distribution based on household wastewater treatment comprises areas with sewage systems connected to a WWTP, areas connected to an RCSF, and areas outside these areas where each residence has a private OWTS (Fig. 1c).

### 2.2 Scenarios for improving sewage disposal rates

#### 2.2.1 Scenario settings

The sewage disposal rate in this study is defined as the proportion of the population connected to one of three types of wastewater treatment system: (1) those connected to a WWTP via a sewage line in their area; (2) those connected to an RCSF; and (3) those using a combined-type OWTS. Direct discharge of greywater is focused and there are no unsanitary situations where toilet water is directly discharged without any treatment. Night soil, generated from unconnected household sewage systems, is collected by the public sector, and single-type systems treat toilet water. We used FY2015 as the base year and determined the present state of the population categorized by type of sewage treatment system (Table S1) and mapped the population distribution based on type of wastewater disposal system (Figs. S1 and S2).

To resolve the problem of untreated greywater discharges, two measures are being promoted: increasing the number of households connected via a sewage line to a WWTP or an RCSF and replacement of single-type OWTSs with the combined type. The three scenarios with measures to improve disposal rates are explained in Table 1. Tables S2–S4 give details of the population in each municipality classified by type of sewage treatment system for each scenario, and Fig. S3 shows the estimated reduction in the population without treatment under each scenario. Scenario B for FY2030 was set assuming the attainment of the medium-term target proposed by Gunma Prefecture [22]. To compare the effects from improved connection rates with those from replacing single-type OWTSs with the combined type, we set up Scenario A, where there are only improvements in the connection rates to a treatment system. Scenario C for FY2040 represents a situation where the connection rates in all municipalities reach 100 %, that is, long-term targets are achieved and direct discharge of greywater is completely eliminated. Regional towns and cities in Japan, including the targeted municipalities, are part of a depopulating society, so we included the reduction of direct discharges through population decline.

**Table 1:** Settings for scenarios toward improvement of sewage disposal rates in Gunma Prefecture.

Scenario	Target year	Sewage disposal rate^a^	Proportion of the population connected to a sewage disposal system
Present state	FY2015	70.5 %	Based on the present situation in FY2015.
Scenario A	FY2030	75.2 %	Connection to a sewage line is promoted; connection rates to WWTPs and RCSFs increase. For each municipality, connection rates to each type of sewage system will reach 90 % if it was less than 90 % initially, and 100 % if it was more than 90 % initially.
Scenario B	FY2030	88.2 %	In addition to the improvements in Scenario A, single-type OWTSs will be replaced with the combined-type. The proportion of the population with OWTSs in each municipality that use a combined-type will increase and achieve the medium-term target by FY2030 as set by Gunma Prefecture.^b^
Scenario C	FY2040	100 %	Sewage disposal rates reach 100 % in all municipalities and achieve the long-term target of Gunma Prefecture^b^ through increasing connection rates to WWTPs and RCSFs and in the proportion of the population using combined-type OWTSs relative to Scenario B.

a Combined value for all municipalities in Gunma Prefecture and four additional adjacent ones.

b Gunma Prefecture [22].

The total sewage disposal rate for all municipalities was set at 70.5 % under present conditions, and this would increase to 75.2 % under Scenario A and 88.2 % under Scenario B (Table 1). This implies that replacing single-type OWTSs with the combined type has a much greater impact on reducing untreated greywater discharge than increasing the connection rate. Although OWTSs have been considered private equipment because they are installed on private lands, the Private Sewerage System Act as revised in 2019 enables local governments to install public OWTSs to promote the replacement of single-type systems. Public OWTS can be installed not only for individual houses, but also for multiple houses as a cooperative. In addition, local governments can now take action against specified existing single-type systems, those with a BOD concentration in the effluent exceeding 90 mg/L or a total coliform count exceeding 3000 number/cm^3^ if they continue to be used as is [23]. In other words, the public sector can intervene both to designate inappropriate single-type OWTSs for continued use and to install combined-type systems as public ones. A decentralized wastewater treatment system is thus transformed into a new system where local government is involved in updating OWTSs.

#### 2.2.2 Estimating BOD discharges

BOD is used to analyze organic pollution loads, and the covered discharge sources are (1) WWTPs treating municipal wastewater, (2) RCSFs treating household wastewater, (3) combined-type OWTSs treating household wastewater, (4) single-type OWTS releasing treated toilet water and untreated greywater, (5) households unconnected to sewage systems releasing untreated greywater, and (6) industrial sector factories. The household wastewater generation rate was set to be 230 L person^−1^ day^−1^, including 50 L of toilet water and 180 L of greywater; BOD loading was set to 18 g person^−1^ day^−1^ for toilet water and 40 g person^−1^ day^−1^ for greywater [24]. OWTSs treat only household wastewater; Table S5 shows their assumed BOD discharge and removal rates. Examples of physicochemical properties of greywater are shown in Table S6. The nitrogen and phosphorus concentrations in greywater were confirmed to be low because the greywater does not contain urine and feces. On the other hand, the greywater contains a lot of organic matters, BOD. An RCSF comprises a flow-rate adjustment tank, anaerobic tank, contact aeration tank, and sedimentation tank, and their removal rates were assumed to be the same as that of a combined-type OWTS, because of the similarity of their processes. Drainage volumes from WWTPs were estimated by adding the volumes of household and commercial wastewater, based on the connected populations and additional urban wastewater, and BOD discharge rates were calculated based on the values in each type of treated water (Table S7). Of the 38 WWTPs, there are 19 plants with a wastewater disposal capacity of more than 10,000 m^3^/day on a clear weather basis, and they use the standard activated sludge method for secondary treatment. The remaining 19 plants are classified as small-scale plants, and 14 of these adopt the oxidation ditch method. None of the WWTPs produce recycled sewage water, so the only tertiary treatment is chlorination; there is no advanced treatment such as ozone treatment. Spatial data for industrial value of shipments were inserted from RIETI [25], and treated water and BOD discharges from factories were estimated by using drainage rates and BOD values for 14 industrial sector categories (Table S8).

Table 2 shows the present status of BOD discharges and for each scenario based on the estimated distribution map (Fig. S4). Under present conditions, BOD discharges from households counted as the “untreated population” were the main discharge source, although the release of untreated greywater was a non-point source. BOD discharge from the untreated population was drastically reduced as the scenarios progressed and became zero in Scenario C by achieving a sewage disposal rate of 100 %. Although the scenarios were designed to include increased sewage-system connection rates, the estimated BOD discharges from WWTPs in FY2030 and FY2040 decreased because of a decline in the regional population. Scenario A is a good example of this trend; total BOD discharges from RCSFs and combined-type OWTSs decreased from the present state even though the connection rate to RCSFs was improved. The main factor responsible for reduced BOD discharges is the conversion from single-type systems to the combined type, and BOD discharges, mainly from combined-type OWTSs, increased from Scenario A to B and from B to C as a consequence of reducing the untreated population.

**Table 2:** Summary of total BOD discharges.

Scenario	Target year	Total BOD discharge (kg/y)			
		WWTP^a^	Households counted as population with sewage treatment^b^	Households counted as population without sewage treatment^c^	Industry^d^
Present state	FY2015	366,813	1,115,663	11,027,790	679,006
Scenario A	FY2030	359,403	971,565	8,290,028	
Scenario B	FY2030		1,532,924	3,808,838	
Scenario C	FY2040	345,120	1,716,874	0	

a Combined discharge in treated water from all WWTPs.

b Distributed discharges from RCSFs and combined-type OWTSs.

c Distributed discharges from three types of households considered without treatment: those not connected to a WWTP, those not connected to an RCSF, and those using a single-type OWTS.

d Discharge on a treated-water basis from all factories; the BOD discharge from industry is set to the same value for all scenarios to allow a focus on differences in sewage disposal rates.

### 2.3 Estimating organic contamination in a river basin

#### 2.3.1 Concentration estimation model

We employed the Standardized Hydrology-based Assessment Tool for Chemical Exposure Load (AIST-SHANEL) model, developed at the National Institute of Advanced Industrial Science and Technology (AIST; [26]). AIST-SHANEL, a version of an unsteady-state analysis model available since 2015, is applicable to chemical risk assessment for any river basin in Japan and comprises a spatial and temporal river flow-rate model and a river water-quality assessment model for chemical substances with a gridded spatial resolution of 250 m. The model was customized in this study to estimate stream-water organic pollution in the target river basin. The distribution of river flow and organic pollution concentration were visualized in maps by ArcGIS Pro.

The model first estimates the river flow rate for each grid cell by applying a one-dimensional hydraulic model that consists of two submodels describing evapotranspiration, snow accumulation, melting processes, and runoff processes for each grid cell [27,28]. Estimated daily flow rates at the most downstream grid cell in the target area were arranged in descending order and a target date was extracted to estimate the 75th percentile. The BOD for a grid cell was simulated by dividing its BOD load by the flow rate, and then BOD under the flow-rate value can be taken as the 75th percentile in ascending order. The target condition for comparing scenarios is preferably a day with relative low flow that tends to be unaffected by non-artificial factors such as the resuspension of bottom sediment due to rainfall. In terms of river concentrations and environmental standards to preserve the living environment in Japan, it is also stipulated that BOD be controlled at the 75th-percentile value.

The customized organic pollution analysis model includes BOD, COD, and suspended solids (SS). The basic formula for the physical dynamics, applied for chemical substances, includes advection, diffusion, settling to the sediment, resuspension to the surface water, mass transfer between the surface water and sediment, and biodegradation [26]. In applying the material balance of pollution load, we excluded some phenomena: nitrification of ammonia nitrogen and subsequent nitrogen reactions, which occur especially in areas polluted by urine and livestock manure; diffusion in sediment, which should mainly be considered in areas where the water is stagnant; and the resuspension of bottom sediment, which occurs especially in wet weather. The formula for kinetic analysis is shown in Eq. (1), with an advection and diffusion model added to settling and biodegradation:

(1) $$\frac{\partial }{\partial t}\left(A∙C\right)+\frac{\partial }{\partial x}\left(A∙U∙C\right)=\frac{\partial }{\partial x}\left(A∙D\frac{\partial C}{\partial x}\right)+W∙A/H-K∙A∙C+L$$

$$W=0,\text{when}\;u^{*}\geq u_{c}^{*}$$

$$W=-w∙C,\text{when}\;u^{*}<u_{c}^{*}\text{,}$$

where *A* is the cross-sectional area of river water (m^2^); *C* is the BOD in the river water (g/m^3^); *U* is the cross-sectional mean flow velocity (m/s); *D* is the diffusion coefficient (m^2^/s); *W* is the settling flux (g/m^2^/s); *H* is the mean water depth (m); *K* is the degradation rate coefficient (1/s); *L* is the inflow load to the river (g/m/s); *w* is the settling rate (m/s); u* is the frictional speed (m/s); and *u*_c_* is the upper frictional speed (m/s).

Surface water temperature was estimated based on the heat balance analysis, and the degradation rate coefficient includes a temperature-dependent property by using the Arrhenius equation (2):

(2) $$K=k\times 1.047^{\left(T-20\right)}\text{,}$$

where *k* is the degradation rate coefficient without correction (1/s) and *T* is water temperature (°C).

Dissolved oxygen (DO) was assumed to be sufficient for biodegradation of organic matter, although an increase of water temperature results in a natural decrease of DO levels, especially in summer, due to the decrease in oxygen concentration at saturation. Each grid cell on the map of estimated distribution of BOD discharge at a spatial resolution of 1 × 1 km was evenly divided into 16 sub-cells and inputted to the kinetic analysis at a spatial resolution of 250× 250 m. Discharged water flows to the nearest river via the drainage channel built into the model and then the BOD inflow load, *L*, was calculated by multiplying amount of BOD discharge by the arrival rate.

The outputted spatial grid data include flow rate, BOD, and stream order, beginning with streams of the first order. The stream order is a positive whole number that indicates the level of branching in a river system [29], and it can be difficult to determine if low order sites are rivers or drainage channels. By overlapping outputted spatial grid data with data for the stream and river channels [30], including class A direct control sections, class A designated sections, class B river sections, and “Others” according to the Japan River Act, the grid data for rivers was extracted for third order streams or higher.

#### 2.3.2 Validation of the model

The model was validated on the basis of Factor *X*, the ratio of an estimated value to the observed value, which falls between 1/*X* and *X*. We validated the river flow rate using observed values at 26 water-level and flow-rate observation sites [31]. Factor 5 was selected because estimates for 83 % of the sites (19 of 23) had *X* values between 0.2 and 5 (Figs. S5 and S6). The river flow-rate model in AIST-SHANEL does not take into account the flow adjustment function of dammed reservoirs. Four sites that fulfilled Factor 10 without satisfying Factor 5 were identified as locations likely affected by flow-rate adjustments from dams. The estimated BOD values were also considered reliable when Factor 5 was satisfied.

The modeled BOD values were verified based on the estimates for present conditions in FY2015 by inputting BOD discharges from municipal, household, and industrial wastewater. The BOD at the upper limit of streams was set to 0.5 mg/L (the general detection limit for public water areas) to include the organic pollution load from basic outflow. The validation of BOD values relied on the observed values (5-day BOD) from 74 sites obtained from a water-quality survey in FY2015 [32], and the 75th percentile was selected to match the estimated values. Multiple iterations of the model calibrated the degradation rate coefficient (*k*) and settling rate (*w*) in a river. We then determined the arrival rate in a drainage channel and consequently set *k* = 0.3 (1/day), *w* = 0.3 (m/day), and arrival rate = 100 %. We did not consider degradation or BOD changes in drainage channels in this study, although the analytical model has that capability, because the main sources of discharges are OWTSs outside a sewage-system area, and water discharged from an OWTS quickly reaches the nearest stream or river.

**Fig. 2:**
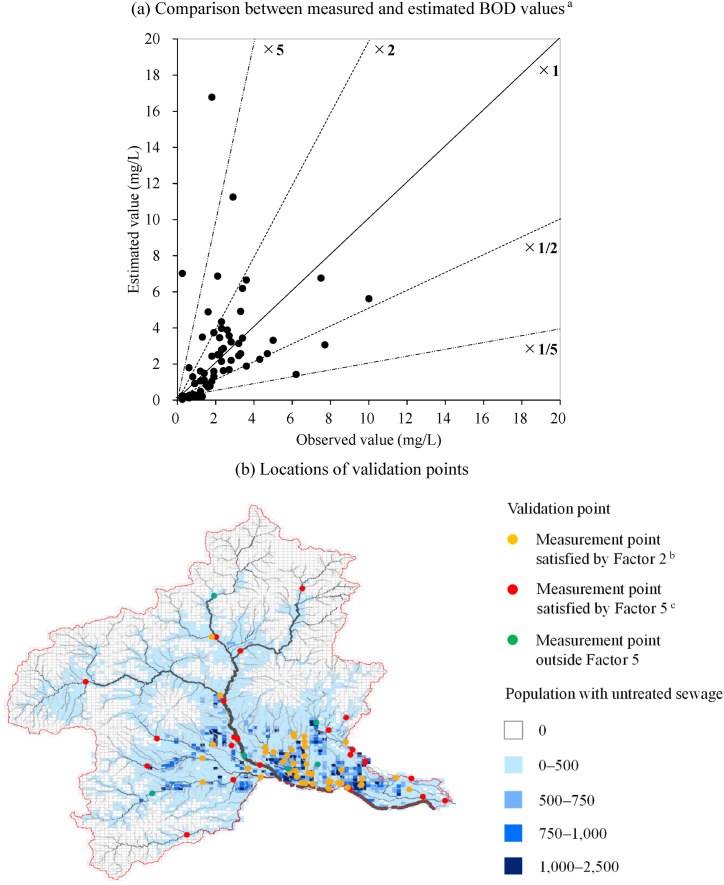
Model validation of BOD estimates. (a) Comparison between BOD values estimated under the “present” conditions (FY2015) by the hydrology-based organic pollution analysis model and observations at 74 sites on the rivers in Gunma Prefecture. (b) Locations of measurement points used to validate the model along with the size of the population without sewage treatment. Spatial resolution in (b) is 1 × 1 km. ^a^ Observed data below the BOD detection limit of 0.5 mg/L are shown assuming their half-value: 0.25 mg/L ^b^ Factor 2 indicates that the ratio of estimated to observed values ranged from 1/2 to 2. ^c^ Factor 5 indicates that the ratio of estimated to observed values ranged from 1/5 to 5.

Fig. 2 shows the results of stream BOD validation for FY2015 calculated using the AIST-SHANEL model and observations at 74 sites. The estimated values are considered reliable by using Factor 5 because 92 % of the sites (68 of 74 sites plotted in Fig. 2a) met this criterion. In a similar matter, 57 % (42 sites of 74 sites) fulfilled Factor 2. Fig. 2b shows the locations of the sites used for validation and which Factor *X* applies. It also gives the characteristics of the sites relative to the surrounding population without sewage treatment, that is, decentralized BOD discharge sources. The accuracy at Factor 2 was confirmed for areas with high volumes of untreated wastewater discharged to a river.

It is important to mention the limitations of the stream BOD estimation model from the aspect of spatial factors. The model does not include emissions from treated effluent from livestock, which is assumed to mainly affect observed BOD through nitrogenous-BOD. The degradation rate and the settling rate in a river are spatially different elements, but are applied to the target area with uniform values. The model does not sufficiently handle spatial variation and uncertainties in the stream purification functions in nature.

### 2.4 Evaluation of ecological status based on BOD and EPT richness

We assessed the ecological status at each site based on the stream BOD as estimated by the hydrology-based organic pollution assessment model. Although Iwasaki et al. [20] reported the association between BOD and EPT richness in Kanagawa Prefecture, which is in the Kanto region of Japan (see Fig. S7), we employed a qualitative classification of ecological status using four classes: class I, good (C≤1.0 mg/L); II, moderate (1.0<C≤3.0 mg/L); III, poor (3.0<C≤5.0 mg/L); and IV, very poor (C>5.0 mg/L). Details about the ecological status for each class are provided in Table 3. The main classification for organic contamination was set at a BOD greater than 3 mg/L, the breakpoint evident from the regression lines in Fig. S7. Class I (good) was set at a BOD of 1 mg/L, 2 mg/L below this threshold and with a tendency to have more than 30 EPT taxa (see Fig. S7). Otherwise, class IV (very poor) was set at a BOD of 5 mg/L, 2 mg/L above the threshold. This level of BOD is the boundary point between beta-medium humidity and alpha-medium humidity (active occurrence of anaerobic decomposition in bottom mud) in the Japanese water-quality grades for terrestrial and aquatic biology [33].

**Table 3:** Classification of ecological status using BOD and EPT richness.

Class	Description	Classified by:	
		BOD (*C*, mg/L)	EPT richness (*S*, no. Of species)
I	Good: No concern about ecological impact of organic pollution on EPT richness.	C≤1.0	S ≥36.71
II	Moderate: Some concern about organic pollution and its impact on EPT richness.	1.0<C≤3.0	6.54<S<36.71
III	Poor: Clear concern about organic pollution and very limited EPT richness.	3.0<C≤5.0	S≅6.54
IV	Very poor: High concern about organic pollution.	C>5.0	S≅6.54

The response of total taxon richness of EPT to BOD, to be employed in this study, is based on field surveys of macroinvertebrates. The WET method for assessing the ecological effects of untreated greywater in a short-term chronic toxicity test complements this validity. Chen et al. [34] conducted the algal growth inhibition tests for treated water from combined-type OWTSs, one from single-type OWTSs, and untreated greywater. High algal growth inhibition was confirmed in the treated water by single-type OWTSs and greywater. A correlation coefficient of nearly of 0.5 was obtained for BOD for greywater, meanwhile, positive correlations were observed for NH_4_–N, non-ionic ammonia, and BOD for treated water from single-type OWTSs.

## 3 Results

**Fig. 3:**
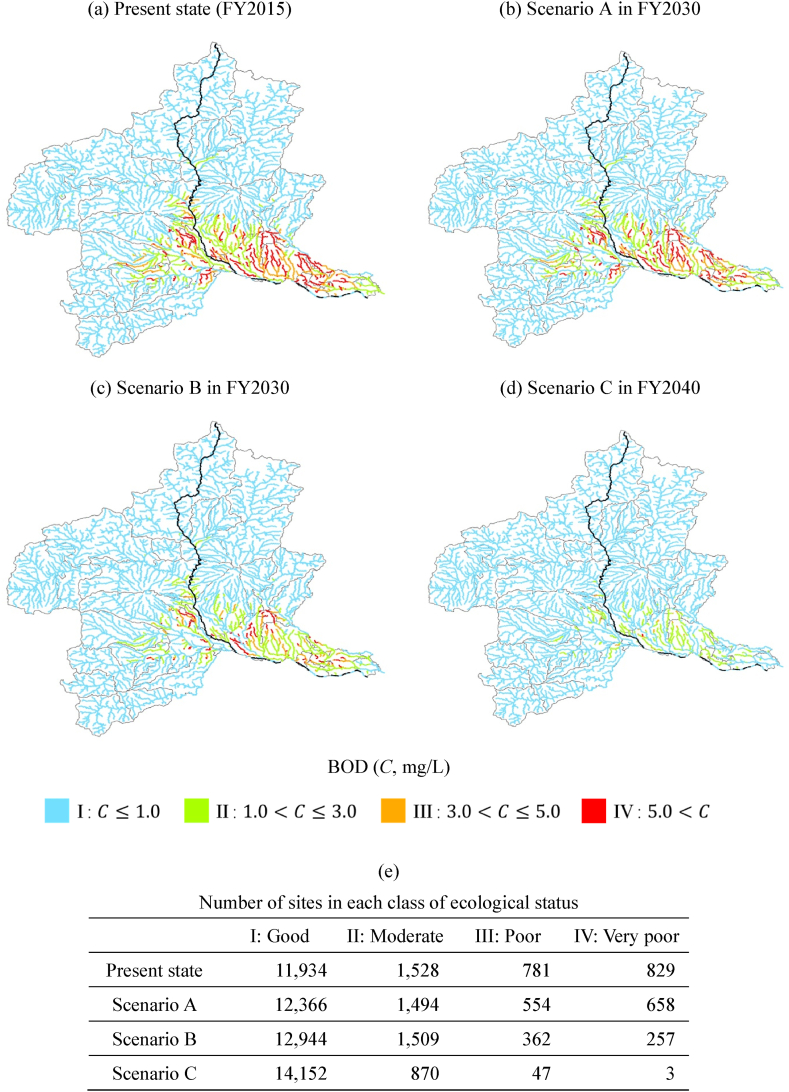
Ecological status of streams in Gunma Prefecture, as determined by estimated BOD under each scenario. The black line shows the main channel of the Tone River. (a)–(d) Estimated ecological status per river site in each scenario: present state, Scenario A, B, and C, respectively. (e) Aggregate values for river sites classified by ecological status in the targeted river basin.

We used the estimated stream BOD values to classify streams by ecological status for each scenario (Fig. 3. 3a: present state, 3b: Scenario A, 3c: Scenario B, and 3d: Scenario C). The effectiveness of the improvements in sewage disposal rates was evaluated by the number of sites with different classifications of ecological status on small and medium-sized rivers in Gunma Prefecture. The number of sites evaluated as in classes III and IV, regarded as organic-polluted sites and aggregated in Fig. 3e, were 781 and 829, respectively (total 1610), under the present state in FY2015. Compared to that state, the number of organic-polluted sites in FY2030 would decrease to 1212 (25 % reduction) under Scenario A, which promotes connections to WWTPs or RCSFs via sewage lines, and to 619 (62 % reduction) under Scenario B, which accelerates upgrades to combined-type OWTSs in addition to the connections promoted under Scenario A. Scenario C would reach 50 class III and IV sites (97 % reduction) in FY2040 by achieving a 100 % sewage disposal rate. Discharges of untreated greywater from non-point sources are major contributors to stream organic pollution in regional towns and cities where improvements in sewage disposal rates are focused, and its elimination has a strong potential to reduce the number of organic-polluted sites.

**Fig. 4:**
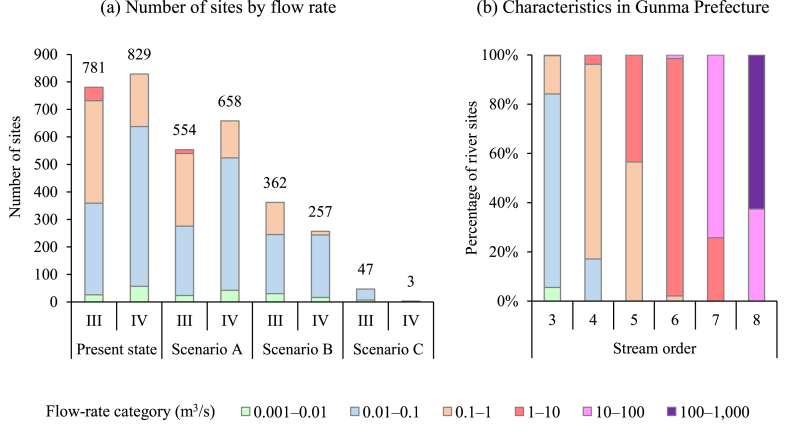
The number of sites in ecological status class III (poor) or IV (very poor), grouped by flow rate. (a) Locations considered organically polluted under each scenario grouped by flow rate. (b) Percentage of sites in each flow-rate range summarized by stream order for small and medium-sized rivers in Gunma Prefecture.

Like GWF, water pollution has an aspect that is evaluated in terms of the volume of freshwater into which it is discharged, and water pollution level is evaluated as the ratio of GWF to the flow rate at the discharge point [8]. The determination of sites as suffering from organic pollution must be considered from the aspect of flow rate, because low volumes of diluting water lead to high BOD values. In addition, flow rate can be treated as one of the factors correlated with EPT taxa, such as average seasonal flow or annual flow [35] or current velocity [19]. Number of sites evaluated as having ecological status classes III or IV were estimated considering flow rates (Fig. 4a). The flow rates classified by stream order at river sites in Gunma Prefecture are summarized in Fig. 4b. Third order rivers have a high proportion of sites where the flow rate is of the order of 0.01 m^3^/s and those of fourth order have a high proportion where flow rate is of the order of 0.1 m^3^/s. Under the present state, discharges of untreated greywater remain heavy, and sites evaluated as class III and IV were mainly where flow rates are on the order of 0.01 and 0.1 m^3^/s. That is, discharges from non-point sources create organic-polluted sites in small branch rivers, not the main river. Tamura et al. [36] constructed a numerical model to estimate aquatic concentrations of an antifungal agent triclosan and an anionic surfactant linear alkylbenzene sulfonate in a small urban stream with no sewer system, Tsumeta River, Tokushima, Japan. These chemicals can be treated as tracer of untreated greywater, and they also showed that river water pollution tended to occur in small and medium-sized rivers. Improvements in sewage disposal rates contribute to the reduction of organic-polluted sites, and under Scenario B, class IV sites are almost eliminated where the flow rate is on the order of 0.1 m^3^/s and most class III and IV sites are where flow rates are on the order of 0.01 m^3^/s. Under Scenario C, only 47 class III sites remain (7 where flow rate is on the order of 0.001 m^3^/s and 40 where it is on the order of 0.01 m^3^/s) along with 3 class IV sites, where flow rates are on the order of 0.01 m^3^/s. Sites with organic pollution are eliminated at sites with flow rates greater than 0.1 m^3^/s.

## 4 Discussion

The ecological status of a river can be assessed by considering whether it runs inside or outside an area with a sewage system. The main source of untreated greywater discharge is the single-type OWTS, but an OWTS itself is a system that preserves stream quality by not only treating wastewater but also performing the purification function of nature on effluent. In addition, the management goals for a river are non-singular; for example, it must generate a rich interaction between people and the river, ensure a rich ecosystem, and preserve the river as a water source. In general, in areas where a sewage system functions to eliminate stormwater from urban areas, more flood control measures are required for the river and the more difficult it becomes to pursue biological conservation.

**Fig. 5:**
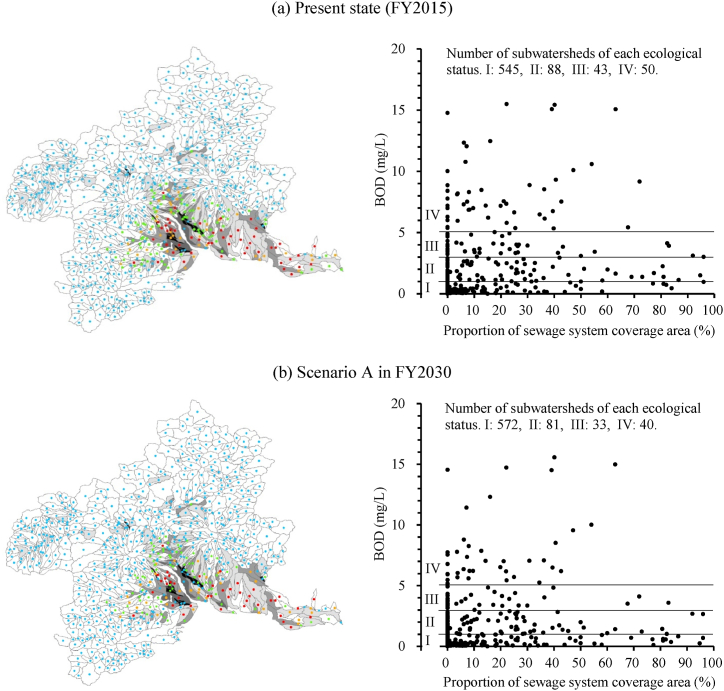

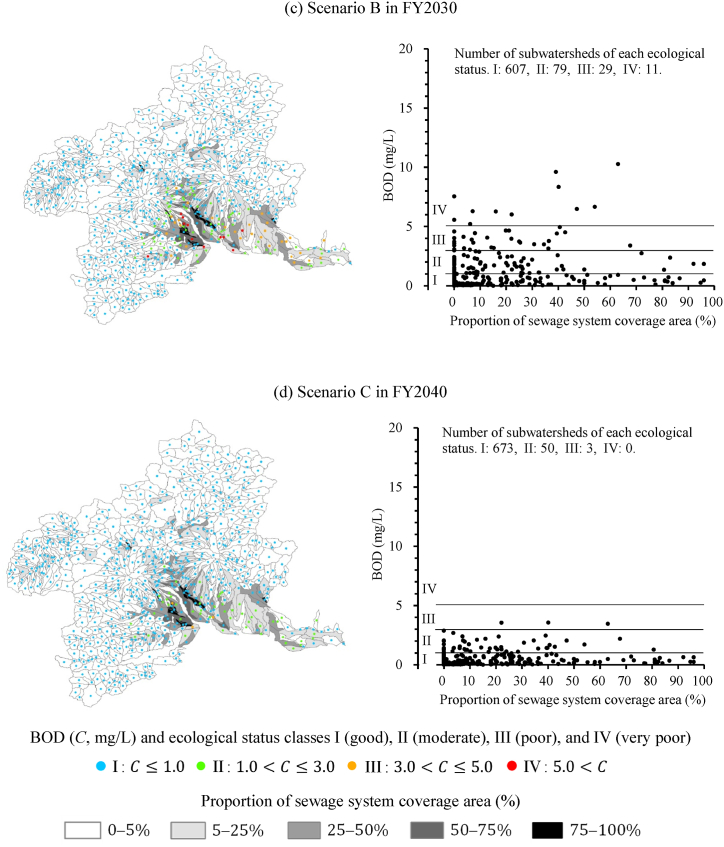
Predicted ecological status of subwatersheds in Gunma Prefecture under each scenario using 75th percentile BOD values, considering the proportion of sewage system coverage area served by a WWTP or an RCSF. (a)–(d) Estimated results in each scenario: present state, Scenario A, B, and C, respectively.

To consider the proportion of an area served by a sewage system, we visualized organic-polluted rivers at the subwatershed level, divided into areas where precipitation drains into rivers and land-use characteristics around rivers can be considered. We estimated the spatial 75th percentile BOD values by using a spatial concentration distribution for each subwatershed [37] and evaluated the ecological status on a subwatershed basis (Fig. 5). We then characterized the land use for each subwatershed by calculating the proportion of the area connected to a WWTP or RCSF. The area was calculated by correcting the mesh data by the proportion of urban land use [38].

The maps in Fig. 5 help us to consider the characteristics of areas where increased sewage disposal rates contribute to improved water quality. Under the present state (Fig. 5a), there are 43 class III and 50 class IV subwatershed areas; only 6 class III and 4 class IV areas have more than 50 % of the sewage system coverage area. In areas with sewage systems, sites with organic pollution are less likely to occur because direct discharge of greywater tends to be farther downstream through a drainage channel. Under Scenario A (Fig. 5b), there are 3 class III and 2 class IV subwatersheds with more than 50 % sewage system coverage area, and there was only a limited effect from improving sewer connection rates. The main factor is the pollution load from users of single-type OWTSs; organic-polluted areas are evident where the proportion of the area served by a sewage system is low, and efforts to ensure a rich ecosystem are required. Scenario B (Fig. 5c), which promotes the conversion to combined-type OWTS, yielded an estimated 29 class III and 11 class IV areas. In particular, the number of class IV areas with less than 50 % sewage-system coverage dropped to nine. On the other hand, this scenario predicted that class III and IV areas would remain under conditions where there is room for improvement in the sewage disposal rate, including areas with a low proportion of sewage-system connections. Scenario C (Fig. 5d), in which the disposal rate reaches 100 %, predicts no more class IV subwatersheds and only three class III areas. An OWTS is a system that preserves stream quality, including performing the purification function of the stream. Scenario C resulted in 50 areas that did not move into class I, but rather into class II. The visualization of the organic-pollution situation at the subwatershed level supports decision-making to ensure a rich river ecosystem.

There is a particular need to update to the combined-type OWTS in subwatersheds with relatively high population densities of OWTS users. On the other hand, 545 subwatersheds were classified as class I under the present state and have no room for organic pollution improvement, mainly due to low population density. This demonstrates the usefulness of being able to identify areas where OWTS renewal would be highly effective in improving pollution loadings. A cooperative OWTS, in which multiple households and up to 50 people use connecting sewage pipes to share a tank, is also an option in villages that are outside the sewage-system area but have a relative high population density. The Private Sewerage System Act revised in 2019 enables local governments to install public OWTSs, including cooperative ones, by covering 90 % of the initial investment. It is necessary to verify the effect of public investment when the local government increases the public expenditure rate and promotes the renewal of OWTSs; a map that visualizes the ecological status helps to select the areas in which to promote the installation of public OWTSs.

Japanese measures to reduce untreated greywater discharges have centered on upgrading to combined-type OWTSs in areas with decentralized wastewater treatment systems, but greywater reuse should also be considered as a useful approach. For example, there should be a focus on onsite treatment and reuse of greywater as toilet flush-water by using biological filtration equipment such as rotating biological contactors and membrane bioreactors, as well as on the environmental partitioning and fate of chemicals that are mainly found in greywater [39]. Treating greywater by nature-based solutions like green walls and green roofs has also received attention, and removal efficiencies have been summarized by Boano et al. [40]. There is a trade-off between water-quality improvement and greenhouse-gas emissions in on-site aerobic tanks, and upgrading to a combined-type OWTS will increase CH_4_ and N_2_O emissions [41]. It is important to consider the improvement of ecological status while balancing greenhouse-gas emission reductions.

## 5 Conclusions

We developed a hydrology-based organic pollution assessment model to estimate the effect of improved sewage disposal rates on organic-polluted sites in light of flow rate and to visualize the potential to improve the ecological status of rivers. There were no organic-polluted sites in rivers with flow rates of 10 m^3^/s or higher; they were found in small and medium-sized rivers (flow rate 0.01–1 m^3^/s) in this case study, because outside of areas with a sewage system, the single-type OWTS is the largest source of BOD load discharge. The organic-polluted sites that were identified in small and medium-sized rivers were influenced by not only pollution load but also by their low flow rates, and visualizing BOD map requires an approach that takes flow rates into account. Given the goals of river management, management to ensure a rich ecosystem is given relative priority outside of areas with sewage systems. In this case study, we attempted to evaluate organic pollution at the subwatershed level and determined that organic pollution occurs in areas with a low proportion of the area served by a sewage system and a high residential population density using single-type OWTSs. The BOD is predicted to decrease with the replacement of single-type OWTSs with the combined type. The visualized distribution of organic-polluted areas supports decision-making to manage a river ensuring a rich ecosystem.

The main purpose of an OWTS is to reduce microbial risk for waterborne pathogens, so treatment of toilet water is prioritized, and some types of OWTS [42], where only toilet water flows into a tank and greywater is directly discharged, are prevalent in some countries, or have been in the past. Selection of a popularized OWTS by considering whether the greywater treatment function is included or not is an important policy decision, because OWTSs are on privately owned property, so they are not easily upgraded. Japan's experience, and the results showing the improvement of stream water quality by upgrading OWTSs focused on greywater treatment, are worth sharing internationally.

From the perspective of the impact of untreated discharges of greywater on aquatic habitats, the ecotoxicity of chemicals contained in personal care products should also be considered [43]. Ecological status based on macroinvertebrates can be evaluated by considering multiple pressures, including land use [44]. This study focused on visualizing the effect of reducing the organic pollution load, but the methodology should be expanded to include the evaluation of ecological status for more complex factors.

## Data availability statement

Data included in article/supplementary material/referenced in article.

## CRediT authorship contribution statement

**Toyohiko Nakakubo:** Writing – original draft, Methodology, Conceptualization. **Midori Kawabata:** Methodology, Investigation, Formal analysis. **Yuriko Ishikawa:** Software. **Yuichi Iwasaki:** Methodology.

## Declaration of competing interest

The authors declare that they have no known competing financial interests or personal relationships that could have appeared to influence the work reported in this paper.
