# Supplementary material for: Modeling the effect of improved sewage disposal rates on ecological status for aquatic organisms in Japan
Source: Heliyon. 2023 Oct 16;9(11):e20943. doi: 10.1016/j.heliyon.2023.e20943 (PMC10618779; doi:10.1016/j.heliyon.2023.e20943)
Supplement: Multimedia component 1 [file mmc1.docx]

Modeling the effect of improved sewage disposal rates on ecological status for aquatic organisms in Japan

Toyohiko Nakakubo ^a,^*, Midori Kawabata ^b^, Yuriko Ishikawa ^c^, Yuichi Iwasaki ^c^

^a^ Division of Sustainable Energy and Environmental Engineering, Graduate School of Engineering, Osaka University, 2-1 Yamadaoka, Suita, Osaka 565-0871, Japan

^b^ Cooperative Major in Human Centered Engineering, Graduate School of Humanities and Sciences, Ochanomizu University, 2-1-1 Otsuka, Bunkyo-ku, Tokyo 112-8610, Japan

^c^ National Institute of Advanced Industrial Science and Technology (AIST), 16-1 Onogawa, Tsukuba, Ibaraki 305-8569, Japan

* Corresponding author: nakakubo@see.eng.osaka-u.ac.jp

**Abbreviations**

EPT: *Ephemeroptera, plecoptera,* and *trichoptera*

OWTS: Onsite wastewater treatment system

RCSF: Rural community sewage facility

WWTP: Wastewater treatment plant

**List of supplementary tables**

Table S1. Municipal populations in Gunma Prefecture classified by type of sewage treatment system under the present state.

Table S2. Municipal populations in Gunma Prefecture classified by type of sewage treatment system under Scenario A.

Table S3. Municipal populations in Gunma Prefecture classified by type of sewage treatment system under Scenario B.

Table S4. Municipal populations in Gunma Prefecture classified by type of sewage treatment system under Scenario C.

Table S5. Settings for biochemical oxygen demand (BOD) discharge rates from households and removal rates for each type of wastewater treatment system.

Table S6. Average water quality results by winter and summer for combined-type OWTS, single-type OWTS, and greywater.

Table S7. Settings for BOD values and volumes of treated water discharged from WWTPs.

Table S8. Settings for BOD values in treated water from industrial sectors.

**List of supplementary figures**

Figure S1. Basic conditions in FY2015 showing the population distribution in Gunma Prefecture by type of sewage treatment system. Spatial resolution: 1 × 1 km.

Figure S2. Basic conditions in FY2015 showing the distribution of the population by type of sewage treatment other than WWTP, RCSF, or combined-type OWTS. Spatial resolution: 1 × 1 km.

Figure S3. Reduction in the population with untreated wastewater in each municipality in Gunma Prefecture under each scenario.

Figure S4. Estimated distribution of BOD discharge under each scenario. Spatial resolution: 1 × 1 km.

Figure S5. Comparison between stream flow rates estimated under the present state on the selected day (3 June 2015) by the organic pollution analysis model and observations at 23 sites.

Figure S6. Locations of points used to validate the flow-rate model, and the results of each validation comparison between the estimated flow rate under the present state on 3 June 2015 and observations by using Factor *X*. Also shown are the locations of dams and their water storage capacities.

Figure S7. Relationship between BOD and total taxon richness of EPT based on a monitoring dataset.

Table S1. Municipal populations in Gunma Prefecture classified by type of sewage treatment system under the present state.

| No. | Municipality | WWTP | | RCSF | | OWTS | |
| --- | --- | --- | --- | --- | --- | --- | --- |
|  |  | Connected population | Unconnected population | Connected population | Unconnected population | Combined type user | Single type user |
| G1 | Maebashi | 223,718 | 8,114 | 23,420 | 5,493 | 37,657 | 38,418 |
| G2 | Takasaki | 259,278 | 14,511 | 3,220 | 805 | 35,001 | 64,718 |
| G3 | Kiryu | 77,131 | 10,418 | 3,963 | 645 | 20,028 | 7,827 |
| G4 | Isesaki | 67,471 | 14,411 | 8,970 | 3,318 | 45,162 | 73,373 |
| G5 | Ota | 73,826 | 22,931 | 12,595 | 4,311 | 68,981 | 58,761 |
| G6 | Numata | 25,917 | 3,669 | 1,307 | 125 | 8,812 | 9,509 |
| G7 | Tatebayashi | 31,796 | 4,336 | 650 | 186 | 26,376 | 15,558 |
| G8 | Shibukawa | 25,045 | 7,737 | 16,350 | 3,662 | 12,320 | 14,876 |
| G9 | Fujioka | 15,094 | 4,741 | 0 | 0 | 25,222 | 21,142 |
| G10 | Tomioka | 8,459 | 3,522 | 1,159 | 464 | 14,434 | 21,117 |
| G11 | Annaka | 12,650 | 6,174 | 0 | 0 | 14,781 | 25,168 |
| G12 | Midori | 9,193 | 3,180 | 730 | 81 | 15,111 | 19,950 |
| G13 | Shinto | 4,464 | 1,824 | 2,269 | 1,798 | 1,296 | 1,163 |
| G14 | Yoshioka | 9,739 | 2,480 | 2,897 | 1,344 | 1,218 | 413 |
| G15 | Ueno | 0 | 0 | 0 | 0 | 1,230 | 0 |
| G16 | Kanna | 0 | 0 | 0 | 0 | 1,151 | 807 |
| G17 | Shimonita | 0 | 0 | 0 | 0 | 3,200 | 4,983 |
| G18 | Nanmoku | 0 | 0 | 0 | 0 | 1,325 | 662 |
| G19 | Kanra | 5,779 | 1,583 | 1,942 | 348 | 644 | 1,740 |
| G20 | Nakanojo | 7,311 | 1,083 | 2,915 | 176 | 3,146 | 1,977 |
| G21 | Naganohara | 1,552 | 775 | 513 | 570 | 936 | 1,171 |
| G22 | Tsumagoi | 3,051 | 339 | 2,384 | 94 | 2,197 | 1,727 |
| G23 | Kusatsu | 4,680 | 0 | 0 | 0 | 987 | 788 |
| G24 | Takayama | 0 | 0 | 1,240 | 531 | 1,538 | 384 |
| G25 | Higashiagatsuma | 1,835 | 334 | 1,655 | 292 | 6,563 | 3,659 |
| G26 | Katashina | 825 | 367 | 324 | 243 | 672 | 2,104 |
| G27 | Kawaba | 2,270 | 568 | 0 | 0 | 278 | 371 |
| G28 | Showa | 0 | 0 | 3,192 | 798 | 1,642 | 1,055 |
| G29 | Minakami | 7,834 | 1,470 | 0 | 0 | 5,910 | 4,210 |
| G30 | Tamamura | 25,274 | 2,653 | 0 | 0 | 1,884 | 5,392 |
| G31 | Itakura | 2,971 | 0 | 0 | 0 | 14,840 | 4,508 |
| G32 | Meiwa | 4,198 | 1,698 | 0 | 0 | 3,359 | 3,772 |
| G33 | Chiyoda | 1,522 | 1,333 | 0 | 0 | 4,065 | 7,683 |
| G34 | Oizumi | 6,566 | 2,909 | 0 | 0 | 13,598 | 11,725 |
| G35 | Ora | 4,023 | 1,902 | 0 | 0 | 10,408 | 15,418 |
| T1 | Ashikaga | 95,014 | 13,822 | 0 | 0 | 13,013 | 27,026 |
| T2 | Tochigi | 77,617 | 9,888 | 0 | 0 | 23,791 | 47,868 |
| T3 | Sano | 76,415 | 5,488 | 0 | 0 | 12,236 | 25,764 |
| T4 | Nikko | 39,960 | 8,653 | 0 | 0 | 23,561 | 11,344 |

Table S2. Municipal populations in Gunma Prefecture classified by type of sewage treatment system under Scenario A.

| No. | Municipality | WWTP | | RCSF | | OWTS | |
| --- | --- | --- | --- | --- | --- | --- | --- |
|  |  | Connected population | Unconnected population | Connected population | Unconnected population | Combined type user | Single type user |
| G1 | Maebashi | 216,508 | 0 | 24,182 | 2,687 | 34,747 | 35,449 |
| G2 | Takasaki | 264,827 | 0 | 3,334 | 370 | 32,576 | 60,232 |
| G3 | Kiryu | 63,911 | 7,101 | 3,642 | 405 | 13,446 | 5,255 |
| G4 | Isesaki | 71,792 | 7,977 | 10,972 | 1,219 | 43,077 | 69,986 |
| G5 | Ota | 86,476 | 9,608 | 14,540 | 1,615 | 57,092 | 48,633 |
| G6 | Numata | 22,094 | 2,455 | 1,159 | 0 | 7,307 | 7,884 |
| G7 | Tatebayashi | 28,319 | 3,146 | 585 | 65 | 22,535 | 13,291 |
| G8 | Shibukawa | 24,176 | 2,686 | 14,214 | 1,579 | 10,018 | 12,097 |
| G9 | Fujioka | 15,703 | 1,745 | 0 | 0 | 20,838 | 17,468 |
| G10 | Tomioka | 8,958 | 995 | 1,211 | 135 | 12,427 | 18,182 |
| G11 | Annaka | 14,810 | 1,646 | 0 | 0 | 12,102 | 20,605 |
| G12 | Midori | 10,054 | 1,117 | 684 | 0 | 12,889 | 17,017 |
| G13 | Shinto | 5,571 | 619 | 3,553 | 395 | 1,255 | 1,127 |
| G14 | Yoshioka | 12,286 | 1,365 | 4,064 | 451 | 1,315 | 445 |
| G15 | Ueno | 0 | 0 | 0 | 0 | 750 | 0 |
| G16 | Kanna | 0 | 0 | 0 | 0 | 642 | 449 |
| G17 | Shimonita | 0 | 0 | 0 | 0 | 1,937 | 3,018 |
| G18 | Nanmoku | 0 | 0 | 0 | 0 | 673 | 336 |
| G19 | Kanra | 5,849 | 650 | 1,773 | 197 | 506 | 1,367 |
| G20 | Nakanojo | 5,982 | 665 | 2,422 | 0 | 2,221 | 1,396 |
| G21 | Naganohara | 1,603 | 178 | 727 | 81 | 666 | 833 |
| G22 | Tsumagoi | 2,881 | 0 | 2,005 | 0 | 1,892 | 1,486 |
| G23 | Kusatsu | 3,546 | 0 | 0 | 0 | 744 | 595 |
| G24 | Takayama | 0 | 0 | 1,328 | 147 | 1,326 | 331 |
| G25 | Higashiagatsuma | 1,395 | 155 | 1,204 | 134 | 4,549 | 2,536 |
| G26 | Katashina | 721 | 80 | 341 | 38 | 445 | 1,392 |
| G27 | Kawaba | 2,064 | 229 | 0 | 0 | 260 | 345 |
| G28 | Showa | 0 | 0 | 2,957 | 329 | 1,445 | 928 |
| G29 | Minakami | 5,995 | 666 | 0 | 0 | 4,174 | 2,974 |
| G30 | Tamamura | 25,521 | 0 | 0 | 0 | 1,711 | 4,896 |
| G31 | Itakura | 2,760 | 0 | 0 | 0 | 7,779 | 2,363 |
| G32 | Meiwa | 5,054 | 562 | 0 | 0 | 2,125 | 2,386 |
| G33 | Chiyoda | 2,445 | 272 | 0 | 0 | 2,980 | 5,633 |
| G34 | Oizumi | 7,775 | 864 | 0 | 0 | 12,654 | 10,910 |
| G35 | Ora | 4,685 | 521 | 0 | 0 | 8,182 | 12,121 |
| T1 | Ashikaga | 85,262 | 9,473 | 0 | 0 | 10,953 | 22,747 |
| T2 | Tochigi | 70,402 | 7,822 | 0 | 0 | 20,448 | 41,143 |
| T3 | Sano | 74,996 | 0 | 0 | 0 | 10,623 | 22,369 |
| T4 | Nikko | 34,120 | 3,791 | 0 | 0 | 17,692 | 8,518 |

Table S3. Municipal populations in Gunma Prefecture classified by type of sewage treatment system under Scenario B.

| No. | Municipality | WWTP | | RCSF | | OWTS | |
| --- | --- | --- | --- | --- | --- | --- | --- |
|  |  | Connected population | Unconnected population | Connected population | Unconnected population | Combined type user | Single type user |
| G1 | Maebashi | 216,508 | 0 | 24,182 | 2,687 | 59,221 | 10,975 |
| G2 | Takasaki | 264,827 | 0 | 3,334 | 370 | 57,397 | 35,411 |
| G3 | Kiryu | 63,911 | 7,101 | 3,642 | 405 | 17,295 | 1,406 |
| G4 | Isesaki | 71,792 | 7,977 | 10,972 | 1,219 | 85,180 | 27,883 |
| G5 | Ota | 86,476 | 9,608 | 14,540 | 1,615 | 92,865 | 12,860 |
| G6 | Numata | 22,094 | 2,455 | 1,159 | 0 | 11,101 | 4,090 |
| G7 | Tatebayashi | 28,319 | 3,146 | 585 | 65 | 30,119 | 5,707 |
| G8 | Shibukawa | 24,176 | 2,686 | 14,214 | 1,579 | 16,933 | 5,182 |
| G9 | Fujioka | 15,703 | 1,745 | 0 | 0 | 34,738 | 3,568 |
| G10 | Tomioka | 8,958 | 995 | 1,211 | 135 | 23,527 | 7,082 |
| G11 | Annaka | 14,810 | 1,646 | 0 | 0 | 27,791 | 4,916 |
| G12 | Midori | 10,054 | 1,117 | 684 | 0 | 19,257 | 10,649 |
| G13 | Shinto | 5,571 | 619 | 3,553 | 395 | 2,069 | 313 |
| G14 | Yoshioka | 12,286 | 1,365 | 4,064 | 451 | 1,481 | 279 |
| G15 | Ueno | 0 | 0 | 0 | 0 | 728 | 22 |
| G16 | Kanna | 0 | 0 | 0 | 0 | 982 | 109 |
| G17 | Shimonita | 0 | 0 | 0 | 0 | 3,196 | 1,759 |
| G18 | Nanmoku | 0 | 0 | 0 | 0 | 871 | 138 |
| G19 | Kanra | 5,849 | 650 | 1,773 | 197 | 1,842 | 31 |
| G20 | Nakanojo | 5,982 | 665 | 2,422 | 0 | 3,490 | 127 |
| G21 | Naganohara | 1,603 | 178 | 727 | 81 | 1,499 | 0 |
| G22 | Tsumagoi | 2,881 | 0 | 2,005 | 0 | 2,965 | 413 |
| G23 | Kusatsu | 3,546 | 0 | 0 | 0 | 1,241 | 98 |
| G24 | Takayama | 0 | 0 | 1,328 | 147 | 1,648 | 9 |
| G25 | Higashiagatsuma | 1,395 | 155 | 1,204 | 134 | 6,207 | 878 |
| G26 | Katashina | 721 | 80 | 341 | 38 | 1,535 | 302 |
| G27 | Kawaba | 2,064 | 229 | 0 | 0 | 605 | 0 |
| G28 | Showa | 0 | 0 | 2,957 | 329 | 2,373 | 0 |
| G29 | Minakami | 5,995 | 666 | 0 | 0 | 5,767 | 1,381 |
| G30 | Tamamura | 25,521 | 0 | 0 | 0 | 5,964 | 643 |
| G31 | Itakura | 2,760 | 0 | 0 | 0 | 8,852 | 1,290 |
| G32 | Meiwa | 5,054 | 562 | 0 | 0 | 3,883 | 628 |
| G33 | Chiyoda | 2,445 | 272 | 0 | 0 | 7,480 | 1,133 |
| G34 | Oizumi | 7,775 | 864 | 0 | 0 | 20,344 | 3,220 |
| G35 | Ora | 4,685 | 521 | 0 | 0 | 13,645 | 6,658 |
| T1 | Ashikaga | 85,262 | 9,473 | 0 | 0 | 24,601 | 9,099 |
| T2 | Tochigi | 70,402 | 7,822 | 0 | 0 | 45,134 | 16,457 |
| T3 | Sano | 74,996 | 0 | 0 | 0 | 24,045 | 8,947 |
| T4 | Nikko | 34,120 | 3,791 | 0 | 0 | 22,803 | 3,407 |

Table S4. Municipal populations in Gunma Prefecture classified by type of sewage treatment system under Scenario C.

| No. | Municipality | WWTP | | RCSF | | OWTS | |
| --- | --- | --- | --- | --- | --- | --- | --- |
|  |  | Connected population | Unconnected population | Connected population | Unconnected population | Combined type user | Single type user |
| G1 | Maebashi | 200,369 | 0 | 24,696 | 0 | 64,378 | 0 |
| G2 | Takasaki | 251,706 | 0 | 3,370 | 0 | 86,136 | 0 |
| G3 | Kiryu | 59,071 | 0 | 3,508 | 0 | 15,163 | 0 |
| G4 | Isesaki | 76,334 | 0 | 11,777 | 0 | 107,458 | 0 |
| G5 | Ota | 92,270 | 0 | 15,119 | 0 | 100,243 | 0 |
| G6 | Numata | 20,916 | 0 | 982 | 0 | 13,033 | 0 |
| G7 | Tatebayashi | 28,110 | 0 | 524 | 0 | 31,803 | 0 |
| G8 | Shibukawa | 22,692 | 0 | 12,939 | 0 | 18,403 | 0 |
| G9 | Fujioka | 15,559 | 0 | 0 | 0 | 33,358 | 0 |
| G10 | Tomioka | 8,425 | 0 | 1,168 | 0 | 26,438 | 0 |
| G11 | Annaka | 14,602 | 0 | 0 | 0 | 27,748 | 0 |
| G12 | Midori | 10,244 | 0 | 598 | 0 | 26,493 | 0 |
| G13 | Shinto | 5,799 | 0 | 3,657 | 0 | 2,192 | 0 |
| G14 | Yoshioka | 14,162 | 0 | 4,469 | 0 | 1,735 | 0 |
| G15 | Ueno | 0 | 0 | 0 | 0 | 556 | 0 |
| G16 | Kanna | 0 | 0 | 0 | 0 | 692 | 0 |
| G17 | Shimonita | 0 | 0 | 0 | 0 | 3,444 | 0 |
| G18 | Nanmoku | 0 | 0 | 0 | 0 | 607 | 0 |
| G19 | Kanra | 5,729 | 0 | 1,735 | 0 | 1,532 | 0 |
| G20 | Nakanojo | 5,471 | 0 | 1,953 | 0 | 2,781 | 0 |
| G21 | Naganohara | 1,434 | 0 | 623 | 0 | 1,172 | 0 |
| G22 | Tsumagoi | 2,511 | 0 | 1,742 | 0 | 2,894 | 0 |
| G23 | Kusatsu | 2,851 | 0 | 0 | 0 | 1,064 | 0 |
| G24 | Takayama | 0 | 0 | 1,275 | 0 | 1,451 | 0 |
| G25 | Higashiagatsuma | 1,220 | 0 | 1,003 | 0 | 5,339 | 0 |
| G26 | Katashina | 598 | 0 | 283 | 0 | 1,356 | 0 |
| G27 | Kawaba | 1,990 | 0 | 0 | 0 | 542 | 0 |
| G28 | Showa | 0 | 0 | 2,852 | 0 | 2,140 | 0 |
| G29 | Minakami | 5,175 | 0 | 0 | 0 | 5,490 | 0 |
| G30 | Tamamura | 22,647 | 0 | 0 | 0 | 5,793 | 0 |
| G31 | Itakura | 2,498 | 0 | 0 | 0 | 8,557 | 0 |
| G32 | Meiwa | 5,170 | 0 | 0 | 0 | 4,013 | 0 |
| G33 | Chiyoda | 2,574 | 0 | 0 | 0 | 7,816 | 0 |
| G34 | Oizumi | 7,846 | 0 | 0 | 0 | 21,761 | 0 |
| G35 | Ora | 4,615 | 0 | 0 | 0 | 17,742 | 0 |
| T1 | Ashikaga | 83,463 | 0 | 0 | 0 | 28,955 | 0 |
| T2 | Tochigi | 70,207 | 0 | 0 | 0 | 53,807 | 0 |
| T3 | Sano | 68,914 | 0 | 0 | 0 | 29,285 | 0 |
| T4 | Nikko | 30,840 | 0 | 0 | 0 | 20,673 | 0 |

Notes for Tables S1–S4:

The resident population distribution map for FY2015, with a resolution of 1 × 1 km, was used by the Japanese national census (Kokusai Kogyo, 2019). The populations living in sewage line management areas with connections to a WWTP or an RCSF, or outside of these areas, were estimated based on urban wastewater infrastructure map in Figure 1. The ratio of the connected population to the unconnected one for each area with sewage lines in FY2015 are from Gunma Prefecture (2020), and the ratio of combined-type OWTS users to single-type users was prepared by MOE-J (2017).

The predicted population distribution maps for FY2030 and FY2040, with a resolution of 1 × 1 km, are from MLIT-J (2018). The connection rates to a sewerage system (WWTP or RCSF) and the proportion of combined-type OWTSs under each scenario were set based on the parameters in Table 1.

Table S5. Settings for biochemical oxygen demand (BOD) discharge rates from households and removal rates for each type of wastewater treatment system.

| Type of system | Discharge source | Influent | | | Effluent |
| --- | --- | --- | --- | --- | --- |
|  |  | Water volume  (L/person/day) ^a^ | BOD load  (g/person/day) ^a^ | Removal rates  (%) ^b^ | BOD emission  (g/person/day) |
| RCSF | Toilet water and greywater | 230 | 58 | 90.0 | 5.8 |
| Combined-type OWTS | Toilet water and greywater | 230 | 58 | 90.0 | 5.8 |
| Single-type OWTS | Toilet water | 50 | 18 | 65.0 | 6.3 |
|  | Greywater | 180 | 40 | 0 | 40.0 |
| Household unconnected to sewage systems | Greywater | 180 | 40 | 0 | 40.0 |

^a^ JSWA (2019)

^b^ MLIT-J (2015)

Table S6. Average water quality results by winter and summer for combined-type OWTS, single-type OWTS, and greywater.

| Measurements | | Treated water by combined-type OWTS | | Treated toilet water by single-type OWTS | | Greywater | |
| --- | --- | --- | --- | --- | --- | --- | --- |
|  |  | Winter  (N = 20) | Summer  (N = 20) | Winter  (N = 20) | Summer  (N = 20) | Winter  (N = 20) | Summer  (N = 20) |
| BOD | (mg/L) | 12 | 7.7 | 23 | 9.0 | 69 | 63 |
| NH_4_-N | (mg/L) | 12 | 4.2 | 62 | 49 | 3.1 | 4.5 |
| NO_2_-N | (mg/L) | 0.97 | 0.09 | 2.9 | 11 | 0.01 | 0.06 |
| NO_3_-N | (mg/L) | 4.6 | 3.8 | 37 | 40 | 0.39 | 1.0 |
| PO_4_-P | (mg/L) | 2.6 | 3.2 | 11 | 17 | 0.38 | 0.35 |
| pH |  | 6.92 | 7.31 | 7.10 | 6.39 | 6.57 | 8.23 |

Chen et al. (2023)

Table S7. Settings for BOD values and volumes of treated water discharged from WWTPs.

| Facility | Volume of treated water discharged in FY2015 (m^3^/day) ^a^ | BOD of treated water (mg/L) ^b, c^ |
| --- | --- | --- |
| Central Gunma WWTP | 129,505 | 3.0 |
| Kiryu WWTP | 15,466 | 0.09 ^d^ |
| Hiratsuka WWTP | 2,304 | 2.3 |
| Tone-bizenzima WWTP | 3,431 | 3.0 |
| Okutone WWTP | 10,312 | 3.0 |
| Nishiora WWTP | 6,588 | 0.09 ^d^ |
| Maebashi WWTP | 25,191 | 4.0 |
| Akagiyamadaido WWTP | 16 | 1.0 |
| Haruna rake WWTP | 337 | 1.4 |
| Akutsu WWTP | 10,845 | 1.8 |
| Jonan WWTP | 24,734 | 1.9 |
| Sakaino WWTP | 15,736 | 2.6 |
| Isesaki WWTP | 16,276 | 3.8 |
| Central No.2 WWTP | 9,837 | 3.0 |
| Central No.1 WWTP | 5,936 | 1.0 |
| Tone WWTP | 398 | 1.4 |
| Shirasawa WWTP | 674 | 2.8 |
| Meiwa WWTP | 988 | 1.7 |
| Tatebayashi WWTP | 13,012 | 3.6 |
| Mizusawa WWTP | 251 | 2.7 |
| Yuzawa WWTP | 1,578 | 2.1 |
| Onogami WWTP | 677 | 0.09 ^d^ |
| Koisawa & Hukiyahara Clean Center | 428 | 0.09 ^d^ |
| Nakanojo WWTP | 1,907 | 1.8 |
| Sawatari WWTP | 71 | 2.0 |
| Shima WWTP | 166 | 1.1 |
| Naganohara WWTP | 643 | 1.0 |
| Tsumagoi WWTP | 974 | 2.0 |
| Kusatsu WWTP | 3,997 | 3.7 |
| Agatsuma WWTP | 544 | 1.9 |
| Kawaba WWTP | 614 | 2.5 |
| Hokubu WWTP | 2,593 | 1.8 |
| Yujuku WWTP | 766 | 12.5 |
| Itakuramachi WWTP | 1,006 | 1.9 |
| Oiwafuji WWTP | 5,632 | 2.0 |
| Ashikaga WWTP | 38,337 | 2.5 |
| Sakanishi multi-unit apartments WWTP | 1,006 | 1.6 |
| Sano WWTP | 31,630 | 2.2 |

^a^ Values estimated in this study.

^b^ JSWA (2017)

^c^ 5-day BOD including nitrogenous-BOD due to non-use of nitrification inhibitor.

^d^ BOD values below the detection limit (1.0 mg/L) were set to 0.09 mg/L.

Table S8. Settings for BOD values in treated water from industrial sectors.

| Industrial sector | Discharge rates  (L/day/million yen) | BOD  (mg/L) ^a, b^ |
| --- | --- | --- |
| Food manufacturing | 67.0 | 9.86 |
| Beverage, tobacco, and feed manufacturing | 49.0 | 7.53 |
| Textile industry | 125.5 | 7.24 |
| Wood and wood-product manufacturing, except furniture | 17.0 | 11.44 |
| Furniture manufacturing | 18.5 | 11.44 |
| Pulp, paper, and processed-paper product manufacturing | 26.0 | 11.44 |
| Printing and printing-related manufacturing | 17.0 | 7.80 |
| Chemical industry | 82.0 | 5.12 |
| Petroleum and coal product manufacturing | 25.0 | 5.03 |
| Plastic product manufacturing | 17.0 | 5.03 |
| Rubber product manufacturing | 16.0 | 5.13 |
| Tanning and fur manufacturing | 10.0 | 9.53 |
| Ceramic and stone product manufacturing | 95.0 | 3.93 |
| Steel industry | 66.5 | 2.71 |
| Nonferrous metal manufacturing | 81.0 | 5.74 |
| Metal product manufacturing | 19.0 | 5.29 |
| General machinery and equipment manufacturing | 13.5 | 5.29 |
| Production machinery and equipment manufacturing | 12.0 | 5.29 |
| Commercial machinery and equipment manufacturing | 15.0 | 5.29 |
| Electronic component, device, and circuit manufacturing | 54.0 | 5.29 |
| Electrical machinery equipment manufacturing | 12.0 | 5.29 |
| Information and telecommunications machinery equipment manufacturing | 7.0 | 5.29 |
| Transportation machinery and equipment manufacturing | 26.5 | 5.29 |
| Other manufacturing | 16.0 | 5.29 |

^a^ 5-day BOD including nitrogenous-BOD due to non-use of nitrification inhibitor.

^b^ Median value for each industrial sector.

Reference: MLIT-J (2015)

| (a) Population with sewage disposal through WWTPs | |
| --- | --- |
| 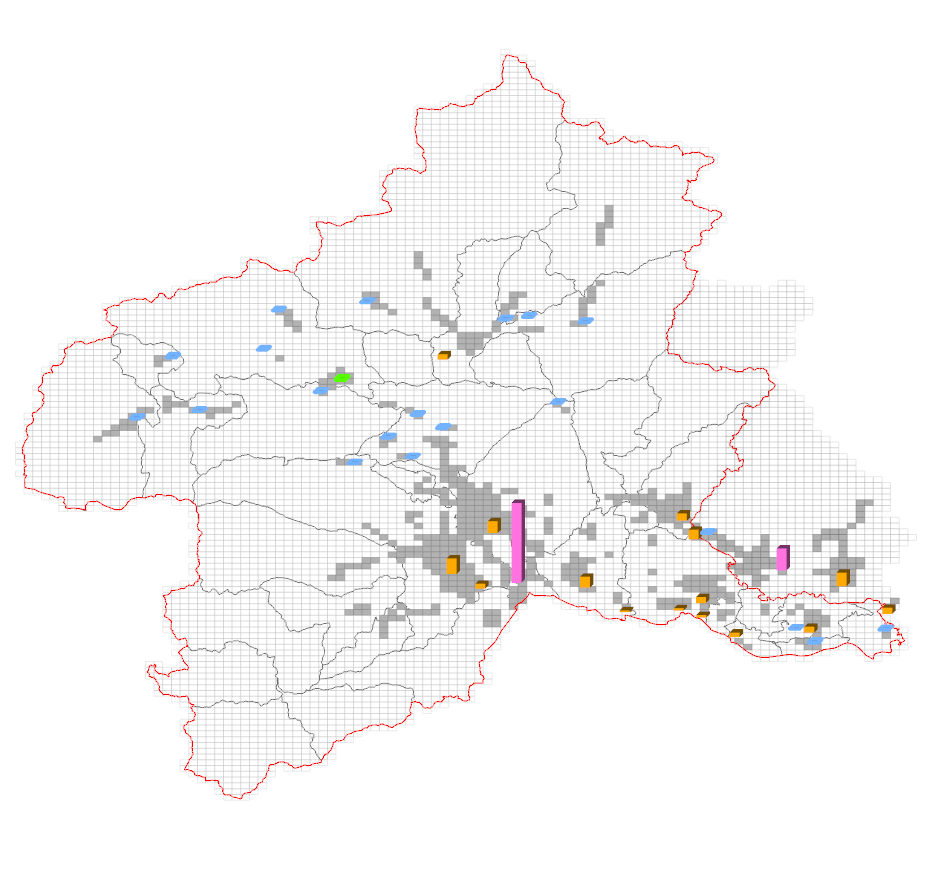 |  |
| (b) Population with sewage disposal by RCSF or combined-type OWTS | |
| 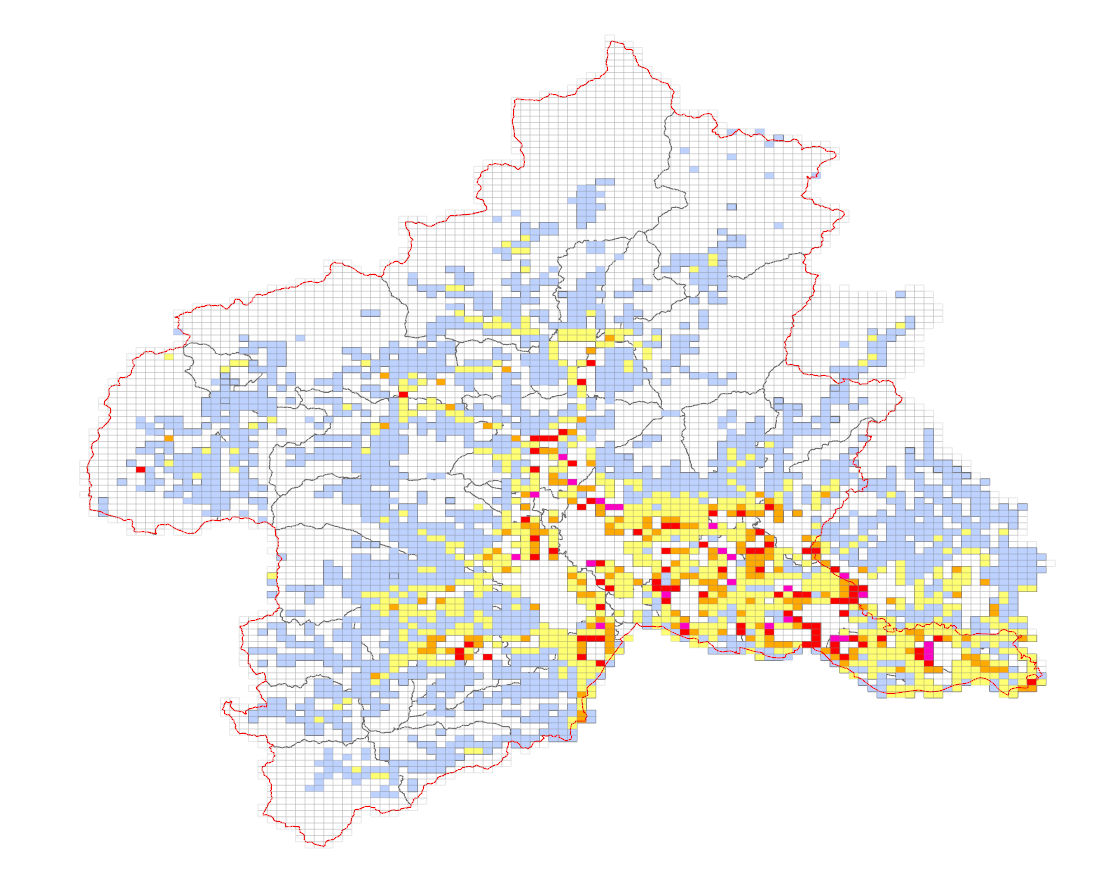 |  |

Figure S1. Basic conditions in FY2015 showing the population distribution in Gunma Prefecture by type of sewage treatment system. Spatial resolution: 1 × 1 km.

| (a) Population not connected to sewage lines in areas served by WWTP or RCSF | |
| --- | --- |
| 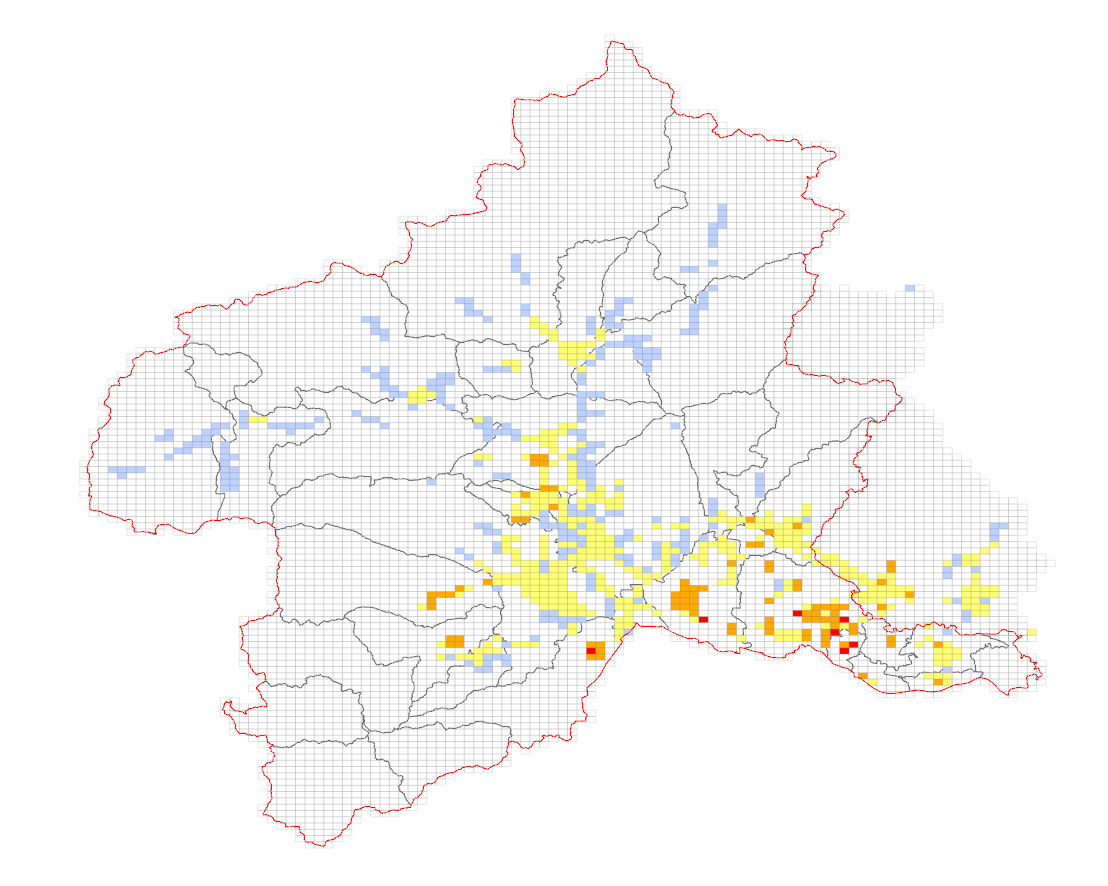 |  |
| (b) Population using single-type OWTS | |
| 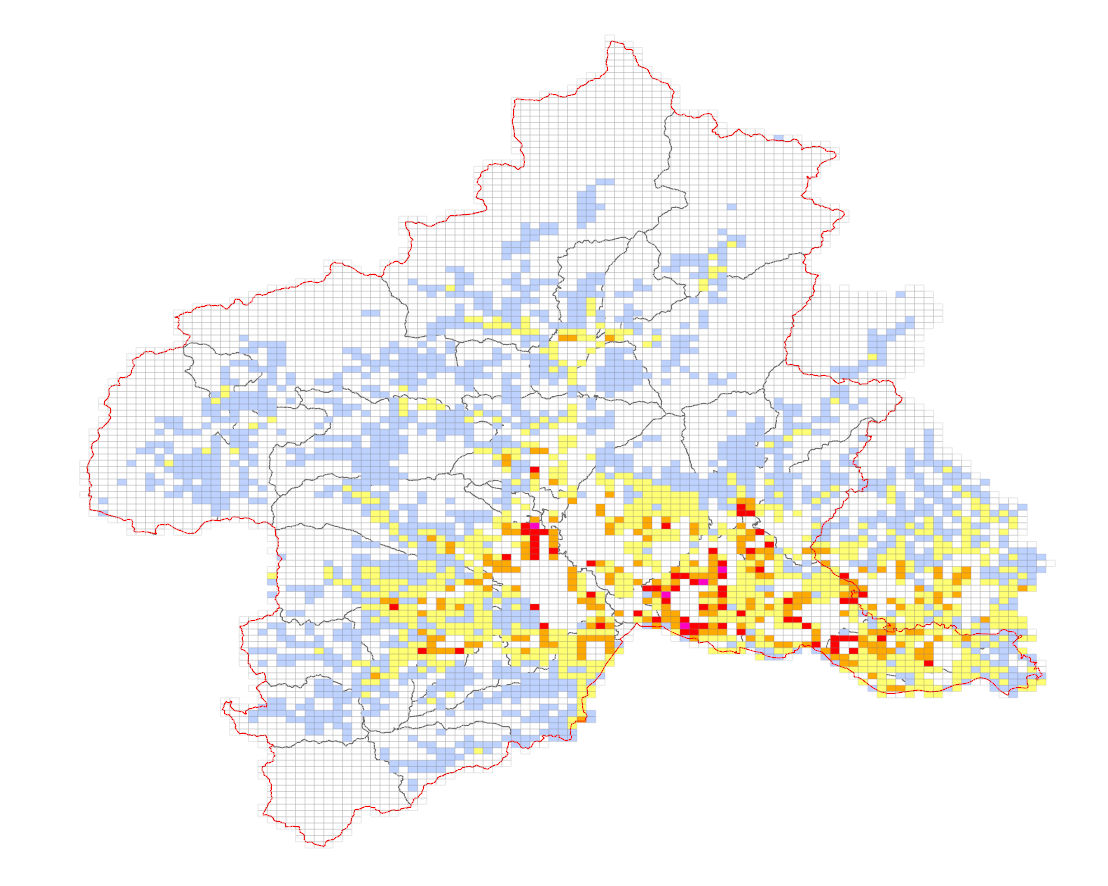 |  |

Figure S2. Basic conditions in FY2015 showing the distribution of the population by type of sewage treatment other than WWTP, RCSF, or combined-type OWTS. Spatial resolution: 1 × 1 km.

| (a) Reduction in untreated population under Scenario B compared to present state |
| --- |
|  |
| (b) Reduction in untreated population under Scenario C compared to Scenario B |
|  |
|  |

Figure S3. Reduction in the population with untreated wastewater in each municipality in Gunma Prefecture under each scenario.

| Present state (FY2015) | Scenario A in FY2030 |
| --- | --- |
| 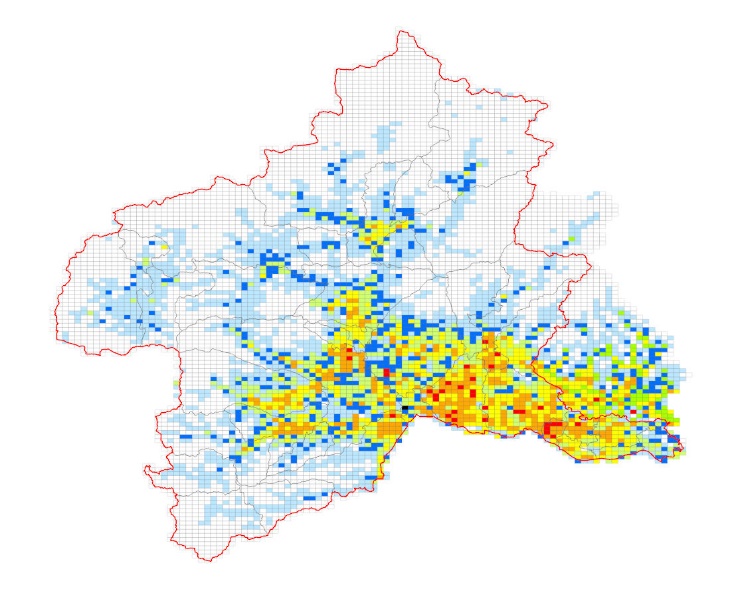 | 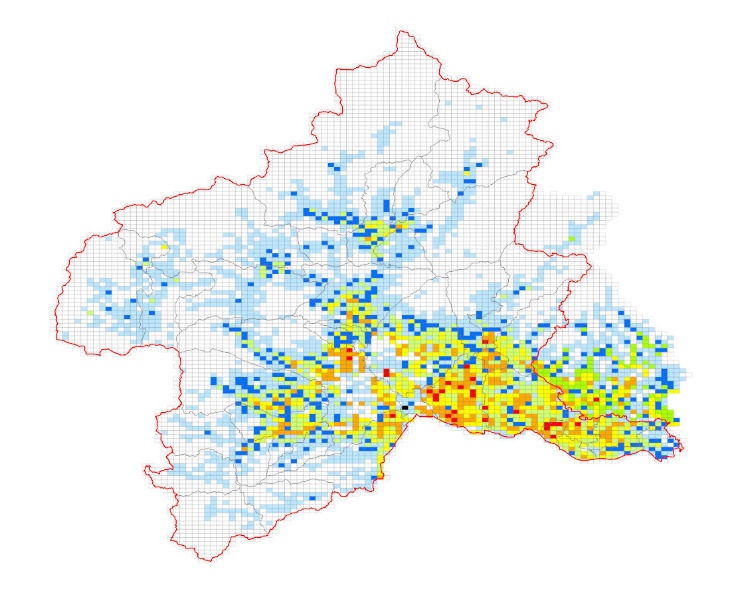 |
| Scenario B in FY2030 | Scenario C in FY2040 |
| 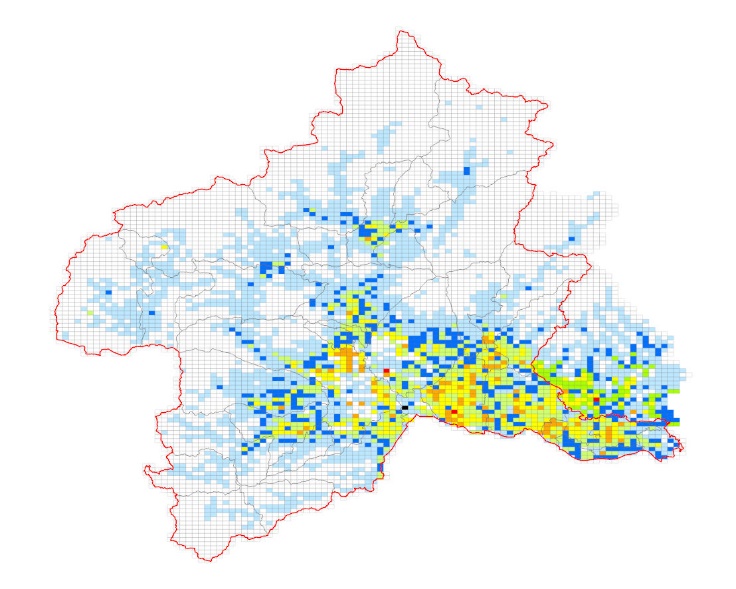 | 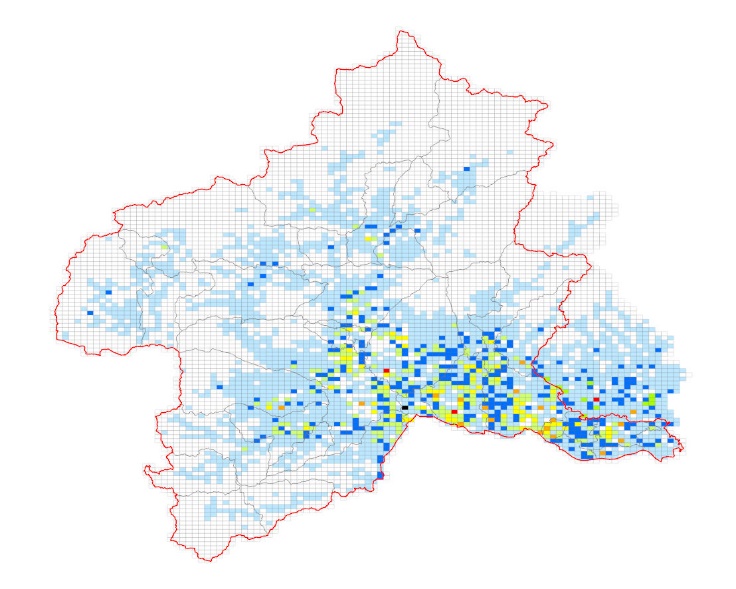 |
| BOD discharge (kg/year)   | |

Figure S4. Estimated distribution of BOD discharge under each scenario. Spatial resolution: 1 × 1 km.

|  |
| --- |

Figure S5. Comparison between stream flow rates estimated under the present state on the selected day (3 June 2015) by the organic pollution analysis model and observations at 23 sites.

Note: Spatial resolution of output mesh is 250 × 250 m. MS, measurement in the main stream; BS, measurement in a branch stream. Factor *X* indicates that the ratio of estimated to observed values ranged from 1/*X* to *X*. Of the 23 sites, 83% (19 sites) have estimates that are satisfied by Factor 5.

|  |
| --- |

Figure S6. Locations of points used to validate the flow-rate model, and the results of each validation comparison between the estimated flow rate under the present state on 3 June 2015 and observations by using Factor *X*. Also shown are the locations of dams and their water storage capacities.

Note: MS, measurement in the main stream; BS, measurement in a branch stream. Factor *X* indicates that the ratio of estimated to observed values ranged from 1/*X* to *X*.

|  | Regression equation:  $S=51.49-14.78\times C, \mathrm{when} C\leq3.04$  $S=6.54, \mathrm{when} C>3.04$  $S$: EPT richness (no. of species)  $C$: BOD (mg/L)  The black line is the piecewise regression model with a zero slope above the breakpoint BOD of 3.04 mg/L (black square) ± 95% confidence interval (error bars) |
| --- | --- |

Figure S7. Relationship between BOD and total taxon richness of EPT based on a monitoring dataset.

Note: Regression analysis is by Iwasaki et al. (2018). The regional-scale monitoring dataset at 32 river sites in Kanagawa Prefecture includes macroinvertebrate monitoring data reported by the Kanagawa Environmental Research Center in 2005 and water quality monitoring data reported by Kanagawa Prefecture in 2003.

**References**

Chen, K., Tanaka, H., Takeda, F., Ebie, Y., Yamazaki, H., 2023. Evaluation of algal growth inhibition of effluents for treated water from Tandoku-syori and Gappei-syori Johkasou using the WET method, J. Water Environ. Technol. 21(3), 141-150. <https://doi.org/10.2965/jwet.22-084>

Gunma Prefecture, 2020. Current penetration condition of sewage system aimed at FY2015, Gunma, Japan. (in Japanese) <https://www.pref.gunma.jp/06/h6610008.html>

Iwasaki, Y., Kagaya, T., Matsuda, H., 2018. Comparing macroinvertebrate assemblages at organic-contaminated river sites with different zinc concentrations: Metal-sensitive taxa may already be absent. Environ. Pollut. 241, 272-278. <https://doi.org/10.1016/j.envpol.2018.05.041>

[JSWA] Japan Sewage Works Association, 2017. Sewerage statistics in FY2015, Tokyo, Japan. (In Japanese) (Japan Sewage Works Association)

[JSWA] Japan Sewage Works Association, 2019. Guideline and commentary for planning and designing wastewater treatment plant, version 2019, Tokyo, Japan. (In Japanese) (Japan Sewage Works Association)

Kokusai Kogyo Co., LTD., 2019. PAREA-Stat. Population Mesh - National census aimed at FY2015, Tokyo, Japan. (in Japanese)

[MOE-J] Ministry of the Environment, Japan, 2017. Survey on actual treatment situation of municipal solid waste - night soil treatment aimed at FY2015. (in Japanese) <https://www.env.go.jp/recycle/waste_tech/ippan/index.html>

[MLIT-J] Ministry of Land, Infrastructure, Transport and Tourism, Japan, 2015. Comprehensive plan for constructing regional sewerage system. (In Japanese) (Ministry of Land, Infrastructure, Transport and Tourism, Japan)

[MLIT-J] Ministry of Land, Infrastructure, Transport and Tourism, Japan, 2018. Population forecast data to 2050 based on national census aimed at FY2015 – Version of a resolution of 1 × 1 km. (in Japanese) <https://nlftp.mlit.go.jp/ksj/gml/datalist/KsjTmplt-mesh1000h30.html>
